# FMRI and intra-cranial electrocorticography recordings in the same human subjects reveals negative BOLD signal coupled with silenced neuronal activity

**DOI:** 10.1007/s00429-021-02342-4

**Published:** 2021-08-07

**Authors:** Alessio Fracasso, Anna Gaglianese, Mariska J. Vansteensel, Erik J. Aarnoutse, Nick F. Ramsey, Serge O. Dumoulin, Natalia Petridou

**Affiliations:** 1 Institute of Neuroscience and Psychology, University of Glasgow, Glasgow, Scotland; 2 The Laboratory for Investigative Neurophysiology (The LINE), Department of Radiology, University Hospital Center, University of Lausanne, Rue Centrale 7, 1003 Lausanne, Switzerland; 3 Department of Radiology, Center for Image Sciences, University Medical Center Utrecht, Heidelberglaan 100, 3584 CX Utrecht, The Netherlands; 4 Department of Neurosurgery and Neurology, UMC Utrecht Brain Center, University Medical Center Utrecht, Heidelberglaan 100, 3584 CX Utrecht, The Netherlands; 5 Experimental Psychology, Helmholtz Institute, Utrecht University, Utrecht, The Netherlands; 6 Spinoza Center for Neuroimaging, Amsterdam, The Netherlands; 7 Experimental and Applied Psychology, VU University Amsterdam, Amsterdam, The Netherlands

**Keywords:** ECoG, Negative BOLD, Positive BOLD, Neuronal activity

## Abstract

Positive blood oxygenation level-dependent (BOLD) responses (PBR), as measured by functional Magnetic Resonance Imaging (fMRI), are the most utilized measurements to non-invasively map activity in the brain. Recent studies have consistently shown that BOLD responses are not exclusively positive. Negative BOLD responses (NBR) have been reported in response to specific sensory stimulations and tasks. However, the exact relationship between NBR and the underlying metabolic and neuronal demand is still under debate. In this study, we investigated the neurophysiological basis of negative BOLD using fMRI and intra-cranial electrophysiology (electrocorticography, ECoG) measurements from the same human participants. We show that, for those electrodes that responded to visual stimulation, PBR are correlated with high-frequency band (HFB) responses. Crucially, NBR were associated with an absence of HFB power responses and an unpredicted decrease in the alpha power responses.

## Introduction

Functional Magnetic Resonance Imaging (fMRI) is a non-invasive technique to measure brain activity via blood oxygenation level-dependent (BOLD) responses. BOLD signals critically depend on the coupling between hemodynamics, metabolic demand, and neuronal activity. Positive BOLD responses (PBR) are the most widely utilized signals. Several animal and human studies have shown a consistent correlation of PBR and local field potentials (Logothetis et al. 2001; Goense et al. 2012a, b), as well as with high frequency broadband responses measured with intra-cranial electrocorticography (ECoG; Hermes et al. 2012; Siero et al. 2014; Gaglianese et al. 2017a, b). However, research has consistently shown that BOLD responses are not exclusively positive. Negative BOLD responses (NBR) can be elicited in specific brain locations during visual and tactile stimulations and tasks (Tootell et al. 1998; Shmuel et al. 2002; Smith et al. 2004; Kastrup et al. 2008; Klingner et al. 2010; Gouws et al. 2014; Fracasso et al. 2018; Jorge et al. 2018). In human primary visual cortex, NBR have been reported adjacent to positive BOLD responses and have been used to measure surround suppression due to stimulation outside the correspondent receptive field (Zuiderbaan et al. 2012). Evidence of NBR has also been shown during mental calculation, within the angular gyrus (Vansteensel et al. 2014).

Notwithstanding the increasing number of studies reporting NBR, the origin of the NBR and its relationship to metabolic and neuronal responses is currently debated, and the mechanisms are less well understood compared to the neurovascular mechanism for the PBR (Devor et al. 2005; Goense et al. 2012a, b). Several competing hypotheses have been proposed to explain the nature of negative BOLD signals. The blood stealing hypothesis was the first to be described by Harel and co-workers (2002). These authors reported that sustained NBR have a vascular origin, independent of local changes in neuronal activity. However, in the last two decades, extensive literature has emerged showing an association between NBR and a corresponding decrease of blood supply and cerebral metabolic rate of oxygen (CMRO_2_), implying a reduction in neuronal activity (Shmuel et al. 2002, 2006; Devor et al. 2008; Boorman et al. 2010). Combining fMRI and electrophysiology experiments, for example, a direct association between NBR and neuronal deactivation has been shown in monkey visual cortex, in terms of a decrease in spiking rate and multiunit activity (Shmuel et al. 2006), and the rat somatosensory cortex, in terms of a decrease in multiunit activity (Boorman et al. 2010). In addition, in humans, an influential study used fMRI to investigate the association between NBR and neuronal deactivation (Shmuel et al. 2002), and showed a coupling between NBR and a decrease in CMRO_2_ in visual cortex. The same results were found for motor cortex and the default mode network (Stefanovic et al. 2004; Lin et al. 2011). Notably, NBR responses have also been associated with decreases in EEG mu power and evoked potential amplitude during median nerve stimulation (8–13 Hz; Mullinger et al. 2014).

Despite the abovementioned evidence for the association between NBR and neuronal deactivation, the coupling mechanism between NBR and neuronal responses is still not yet fully resolved, as multiple mechanisms could be responsible, as pointed out by Goense and colleagues (2016). Moreover, a cautionary note is necessary, since the NBR observed by fMRI in humans are difficult to relate to corresponding neuronal activity using non-invasive electrophysiological recordings, such as EEG or MEG, due to the differences in spatial and temporal resolution between the techniques (Shmuel et al. 2007; Hedrich et al. 2017, see also Fracasso et al. 2021).

Electrocorticography is a neural signal recording technique that is used clinically to determine the seizure onset zone in patients with epilepsy. Since ECoG electrodes are placed sub-durally, this technique provides a unique opportunity to study human brain function in healthy tissue (accessible adjacent to pathological tissue), since it allows for a direct measurement of electrical activity from neuronal populations located directly under the electrodes at the grey matter surface. As such, ECoG provides high spatio/temporal resolution, enabling a more direct comparison with fMRI measurements. Furthermore, ECoG recordings comprise information of both neuronal oscillatory activity as well as changes in high frequency broadband (HFB) power, which has been suggested to be directly associated with spiking activity (Miller et al. 2009).

Here, we investigated the relationship between NBR and neuronal population activity using fMRI and ECoG measurements in the human visual cortex. We used a visual stimulation paradigm consisting of a unilateral section of a moving dart-board pattern (Tootell et al. 1998; Gouws et al. 2014), which is known to elicit two separated responses: PBR on the contralateral visual cortex and NBR on the ipsilateral visual cortex. We aimed at characterizing the association between the ECoG signal and the BOLD signal from the same cortical locations for each participant.

We observed a positive correspondence between PBR and HFB responses, as well as a negative linear correspondence between PBR and alpha responses, consistent with the idea that alpha activity reflects functional inhibition (Jensen and Mazaheri 2010; Palva and Palva 2011). Moreover, we show that NBR is associated with an absence of power increase of HFB activity and a decrease in alpha power.

## Materials and methods

### ECoG

Two patients underwent implantation of subdural electrode grids (Adtech, Racine, USA, interelectrode spacing: 1 cm, electrode diameter: 2.3 mm; Data recording system for participant 1 (P1): 128 channels, 512 Hz sampling rate, 0.15–134.4 Hz bandpass filter, Micromed, Italy; Data recording system for participant 2 (P2): 128 channels, 2048 Hz sampling rate, 0.15–500 Hz bandpass filter, Micromed, Italy) to determine the site of epileptic foci for the purpose of possible surgical removal of the epileptogenic tissue. Implanted grids extended to healthy tissue in the occipital pole of the left hemisphere for P1 and the right hemisphere for P2. The patients gave written informed consent to participate in the study in compliance with the Declaration of Helsinki 2013. The experimental protocol was approved by the medical research ethics committee of the UMC Utrecht.

### fMRI

The two participants underwent pre-operative and post-operative fMRI scanning, respectively. Data were acquired at a Philips Achieva 3T scanner (Philips Healthcare, Best, Netherlands), using a 32-channel head coil and 3D PRESTO (Neggers et al. 2008) with the following parameters: FOV 99X256X256mm, resolution of 3 mm isotropic, 33 slices, flip angle: 10°, TE/TR: 38.7/27 ms. Functional volumes were acquired every 810 ms (Neggers et al. 2008), and run duration was 3 min. Anatomical T1-weighted (T1-w) data were acquired using an 3D MPRAGE sequence (number of excitations per inversion 180; TR/TE 10 ms/4.6 ms; flip-angle 8°; FOV 240 × 240 × 160 mm; 200 slices, 0.8 mm isotropic voxel size; total scan duration 602 s).

### Stimuli

**Fig. 1:**
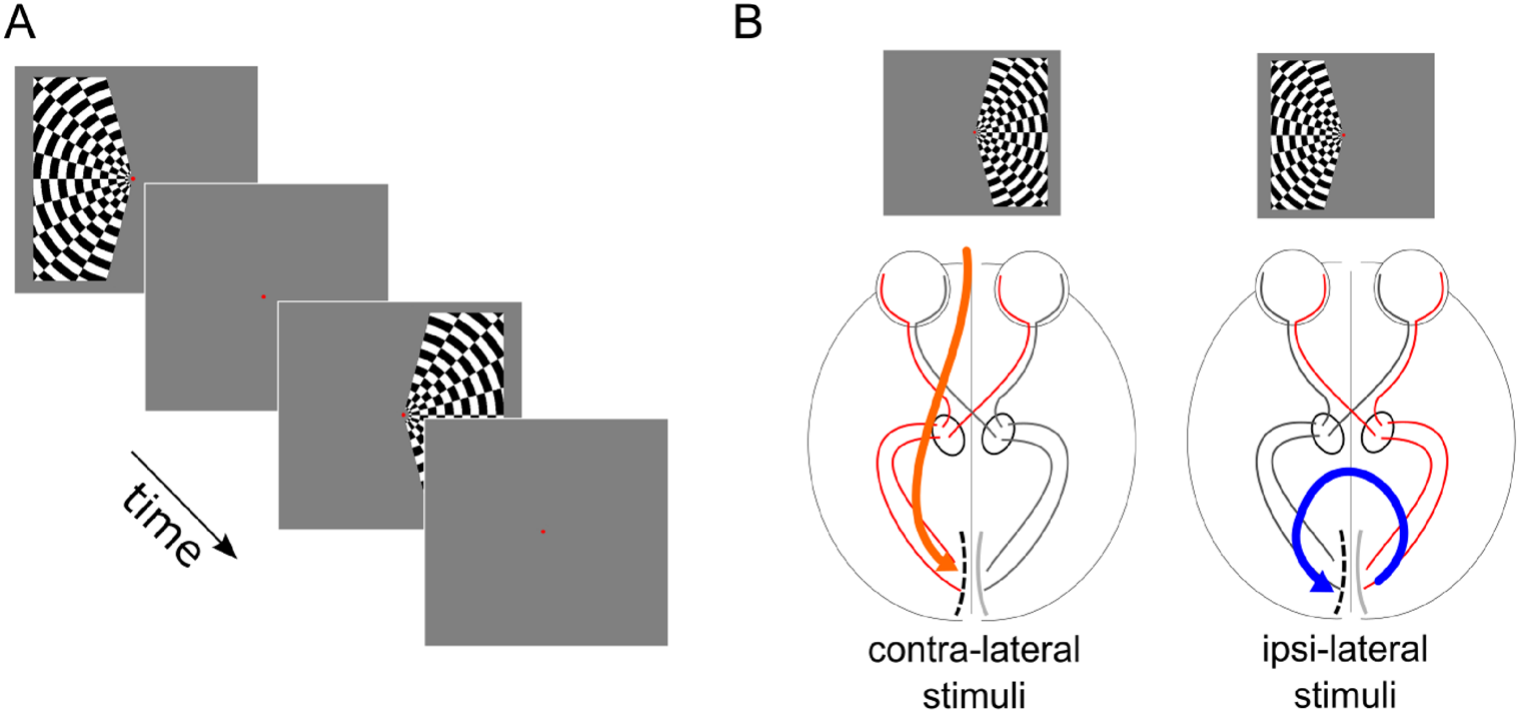
Visual paradigm. Panel **A**: left or right visual hemifields were stimulated by a contrast-defined lateralized dart-board pattern, alternated with periods of gray screen, which constituted the baseline condition (Tootell et al. 1998; Gouws et al. 2014; Fracasso et al. 2018). Panel **B**: participants were asked to fixate on the center of the screen and maintain stable fixation on the red center dot. The alternate segments of the pattern moved in opposite radial directions and the motion direction changed unpredictably. In participant 1 (P1) the electrode grid was placed on the left hemisphere (see dashed line in the sketched primary visual cortex in panel **B**); in participant 2 (P2) the grid was placed over the right hemisphere (not shown). Thus, the ipsi-lateral and contra-lateral conditions were associated with opposite hemispheres in the two participants. The ipsi-lateral and contra-lateral conditions were defined with respect to the placement of the ECoG grid. In the example above (P1, ECoG grid in the left occipital pole) the contra-lateral stimuli response is located in the right hemifield, the ipsilateral stimuli response in the left hemifield. The opposite mapping occurred for P2, where the ECoG grid was placed on the right occipital pole

Avoiding a central circular region (0.4° of visual angle) and displaced by 20° of polar angle from the vertical meridian (Fig. 1A). The stimulus radius was 10° of visual angle. This size (10° of visual angle) was computed based on the distance between the participant’s point of view and the screen, as well as the screen size. 10° were determined by the fMRI environment. We could go higher when measuring ECoG but we were constrained by the fMRI environment. The same stimulus properties were adopted for ECoG and fMRI measurements. The dart-board consisted of a rectangular grid with a spatial frequency of 0.5° of visual angle. Each spoke of the dart-board pattern moved coherently in opposite radial directions with a temporal frequency of 10 Hz. Each stimulus lasted for 810 ms and left and right visual field stimulation was alternated with an interleaving 15 s baseline (gray screen). The visual stimulus in the ECoG setting was identical to the fMRI paradigm, but stimulus duration was shorter. For the ECoG recordings, each stimulus lasted 500 ms with an inter-stimulus interval of 2 s. The participants fixated on the center of the screen and were instructed to maintain stable fixation. Different stimuli and baseline durations were adopted for the fMRI and ECoG measurements. This is due to the different duration of the vascular and neuronal responses measured by two techniques, respectively. fMRI is characterized by a relatively slow hemodynamic response function that can take up to 20 s to return to baseline after a short impulse stimulation (Glover 1999). On the other hand, direct neuronal recordings, such as ECoG measurements, are characterized by a rapid response to short visual stimulation (few ms) and a rapid return to baseline (Gaglianese et al. 2017a, b). 12 stimuli were presented during each fMRI run, 6 on the left hemifield and 6 on the right hemifield. 30 stimuli were presented during one ECoG run, 15 on the left hemifield and 15 on the right hemifield. In P1 we acquired the fMRI run before the ECoG run, vice versa for P2.

### ECoG—pre-processing

Electrodes with epileptic artifacts, as determined by trained neurologists, were removed from further analyses, and signals were re-referenced to the common average of all remaining electrodes. For each participant, power spectral density (1–134 Hz) was estimated for each trial every 1 Hz by Welch’s periodogram, averaging with a 1 s window. Active trials were defined as the 500 ms of visual stimulation during contralateral or ipsilateral conditions. Rest epochs started 500 ms after the stimulus offset and lasted 1 s. Mean responses in the high frequency band (HFB; 65–95 Hz), alpha band (9–13 Hz) and beta band (14–30 Hz) (Hermes et al. 2012; Gaglianese et al. 2017a, b) were extracted for both conditions for further statistical analysis.

### ECoG—analysis

Electrodes exhibiting significant responses for the contralateral condition were selected by statistically comparing the mean responses in the HFB frequency range to the mean power of the rest epochs (paired *t* test, *p* < 0.05 and average spectral power difference > 0). Overall, following the described criteria, *n* = 33 and *n* = 27 electrodes were selected in in P1 and P2, respectively. In addition, for each selected electrode, we computed mean responses in alpha and beta power frequency band for both contralateral and ipsilateral conditions. The responses of each frequency band were converted to *z*-scores by subtracting the mean response across rest epochs from the mean responses (averaged across trials) for each condition and dividing by the standard deviation of the mean of the rest epochs. Significance across all selected electrodes with respect to the null hypothesis of no response was tested using *t* tests for each frequency band (*p* < 0.05, Bonferroni corrected).

In addition, to assess the mean HFB response over time during visual stimulation we filtered the data between 65 and 95 Hz using a 3rd order Butterworth filter in two directions (Hermes et al. 2012) and we calculated the smoothed log power of the analytic amplitude using the Hilbert transform.

### ECoG—control analysis

To assess the robustness of the results, we varied the frequency range of the measured HFB power changes in the contralateral and ipsilateral visual stimulation during ECoG measurements. We opted for three different frequency ranges: low-gamma (31:64 Hz) high-gamma (65:130 Hz), and all-gamma (31:130 Hz). We computed the number of electrodes exhibiting significant responses for the contralateral condition within each frequency range (paired *t* test, *p* < 0.05 and average spectral power difference > 0). Moreover, within each frequency range, we assessed the response HFB response in the ipsilateral condition. Significance across all selected electrodes with respect to the null hypothesis of no response was tested using *t* tests for each frequency range (*p* < 0.05, Bonferroni corrected).

### fMRI—pre-processing

Functional data was motion corrected using the function 3dvolreg in AFNI (https://afni.nimh.nih.gov, Cox., 1996). The average motion corrected functional volume was computed and used as a reference for co-registration with the T1-w anatomical image. Co-registration was performed using the function 3dAllineate in AFNI, with mutual information as cost function.

### fMRI—analysis, global response

**Fig. 2:**
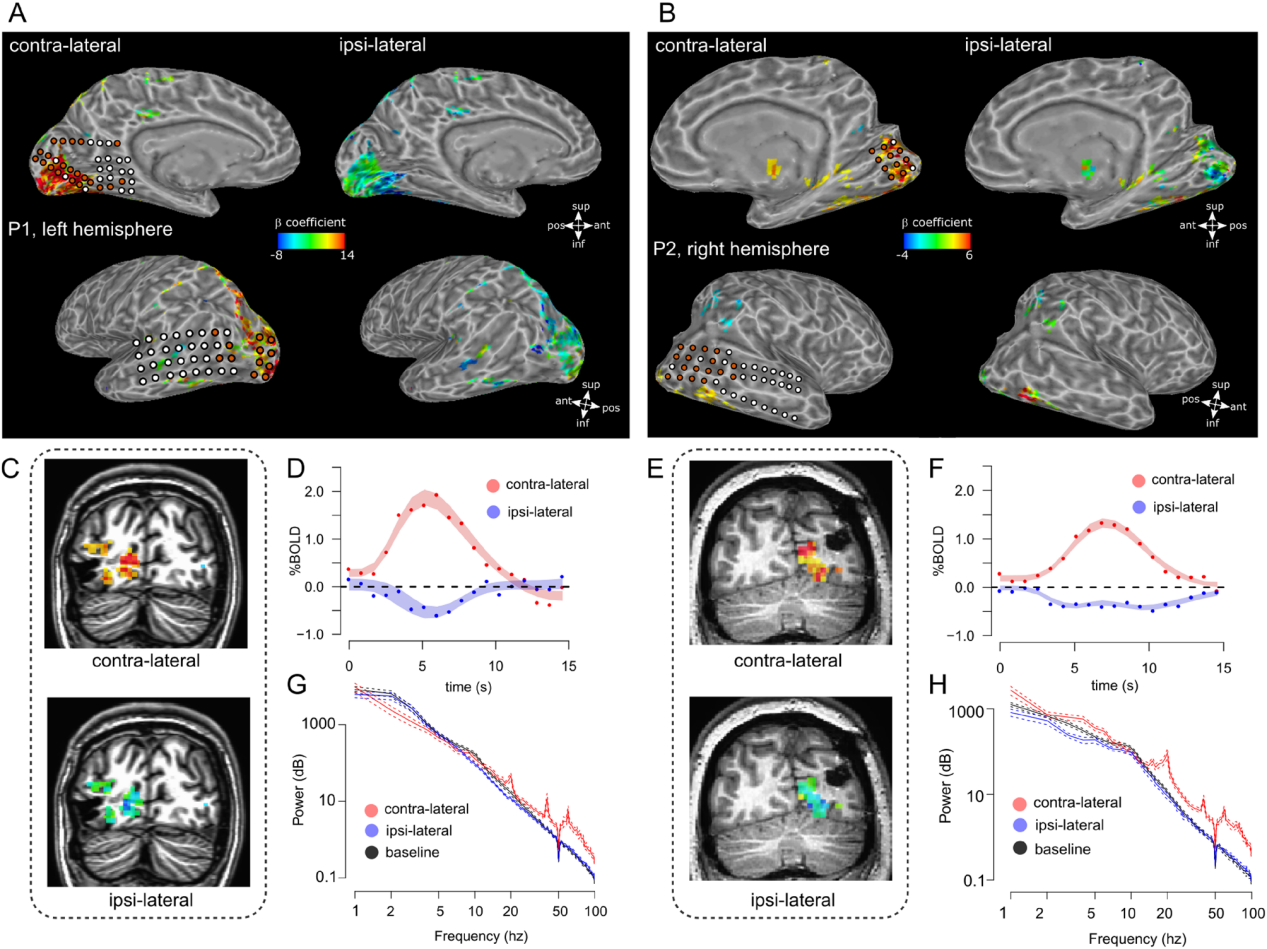
BOLD response (global hrfs see “fMRI—analysis, global response”) and average power spectra in the contra-lateral and ipsi-lateral stimulus conditions for the selected electrodes (see “EcoG—analysis”); panels **A**, **B** BOLD fMRI activation maps on the surface rendering of the brain for the two participants. Superimposed circles indicate the location of the implanted electrodes; orange circles are the electrodes exhibiting significant HFB responses to the contra-lateral stimulation for each participant which were selected for analysis (*n* = 33 and *n* = 27 electrodes in P1 and P2, see “ECoG—analysis”). The maps in panels **A**, **B** shows locations selected based on the contrast of contra-lateral minus ipsi-lateral condition (*T* values > 2, which corresponds to *p* < 0.05). In the contra-lateral condition, we plot the *β* coefficient associated with the contra-lateral condition minus baseline contrast (see legend in panels **A**, **B**). In the ipsi-lateral condition the same activation map is used, plotting the *β* coefficient associated with the ipsi-lateral condition minus baseline (see legend in panels **A**, **B**). Panel **C**: coronal slice, P1, contra-lateral and ipsi-lateral BOLD fMRI activation maps for one example slice. Activation maps represent signal amplitude (*β* coefficient, cluster > 20, threshold by *t*-statistic > 2, *p* < 0.05, uncorrected, contra-lateral: contra-lateral condition minus baseline contrast; ipsi-lateral: ipsi-lateral condition minus baseline contrast). Panel **D**, red curve, average estimated global hemodynamic responses (global HRF see “fMRI—analysis, global response”) for voxels responding significantly to the contra-lateral condition (cluster > 20, threshold by *t*-statistic > 2, *p* < 0.05, uncorrected), shaded area indicates 95% confidence interval of bootstrapped HRF fits (see “fMRI—analysis, global response”). Panel **D**, blue curve, average estimated global HRF for the same voxels for the ipsilateral condition. Panels **E** and **F**, same as **C** and **D**, for P2. Panel **G**, ECoG data: average power spectra across all selected electrodes for P1 (see “ECoG—analysis”). Separate lines report the measured spectra for baseline (gray screen), contra-lateral and ipsi-lateral conditions (error bars indicate ± 1 standard deviation). Panel **H**, same as panel **G** for P2

Functional data was analyzed in native space with a standard GLM using a canonical hemodynamic response function (HRF). To extract the shape of single voxel HRFs a set of basis functions was fit to each voxel via the 3dDeconvolve function in AFNI. Voxels were selected for further analysis based on the contrast of contra-lateral minus ipsi-lateral condition (*T* values > 2, which corresponds to *p* < 0.05, uncorrected, see Fig. 2C–F, this value was only used for voxel selection to extract the global HRFs reported in Fig. 2D, F). We performed a bootstrapping analysis of all the selected voxels to estimate the shape of the contra-lateral and ipsi-lateral HRF (Fig. 2D, F; Efron and Tibshirani 1994). It is important to note that these HRFs are representative of *all* the selected voxels based on the contrast between contra-lateral and ipsi-lateral responses and *do not* reflect the response of a single voxel or below a single electrode, for this reason we refer to *global* HRF (see Fig. 2, caption). Selected voxels were bootstrapped 5000 times with replacement. For each iteration, the average HRF was computed for the contra-lateral and ipsi-lateral stimulation conditions. The HRFs obtained were fit using a double-gamma hemodynamic response function with three free parameters: amplitude, delay of the response relative to onset and delay of the undershoot relative to onset. Fitting was performed using non-linear regression in R (R Development Core Team 2010) and we computed the 95% bootstrapped confidence intervals of the fit (Fig. 2D, F).

### ECoG–fMRI co-localization and local HRF response

The location of each implanted electrode was determined on a post-operative CT scan using an automatic clustering detection algorithm as described in Branco et al. (2018). The T1-w anatomical images were segmented automatically using FreeSurfer (https://surfer.nmr.mgh.harvard.edu). White matter and pial surfaces were generated in Freesurfer and then imported in SUMA (https://afni.nimh.nih.gov). After co-registering the CT scan to the anatomical scan, thereby bringing ECoG and fMRI data in the same space, electrode coordinates were projected on the closest vertex on the cortical surface in the anatomical T1-w scans.

For each participant, electrodes responding to the task were selected based on ECoG HFB responses to contralateral stimulation. Power increase in the HFB has been directly associated to spiking activity (Fries et al. 2007; Miller et al. 2009; Hermes et al. 2015), and it provides a precise measure to localize active brain regions during visual stimulation.

To match the ECoG measurements with the BOLD fMRI data, the fMRI signal under each selected electrode was extracted from gray matter voxels within 3 mm (radius) around the electrode center.

**Fig. 3:**
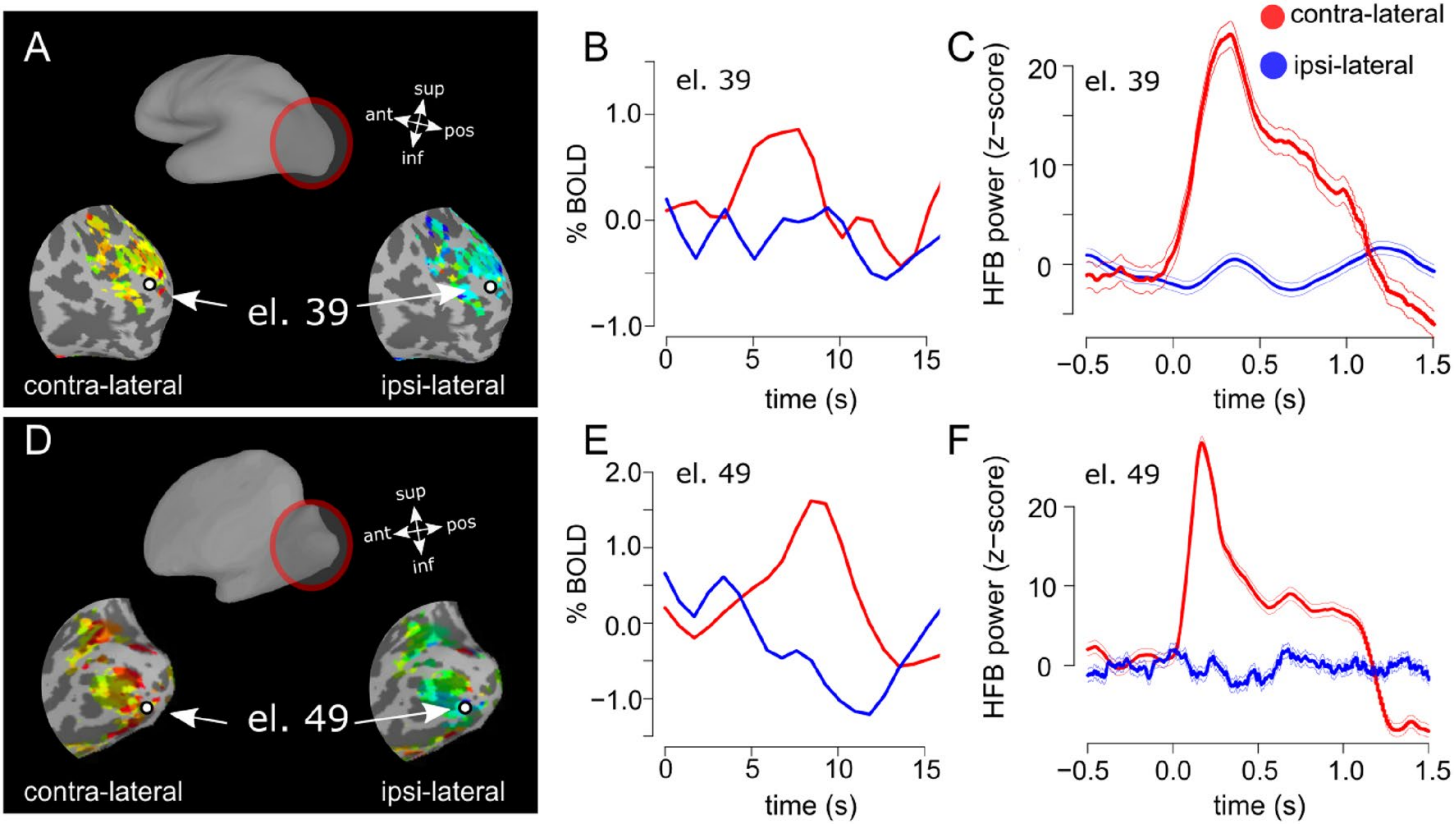
Representative single electrodes, BOLD and HFB power. Panel **A**, electrode location over the reconstructed and inflated brain surface superimposed to contra-lateral and ipsi-lateral fMRI activity, P1. Panel **B**: HRF for locations corresponding to the same representative selected electrode, for contra- and ipsi-lateral conditions (see “ECoG–fMRI co-localization and local HRF response”). Panel **C**, HFB responses for the representative electrode. An increase in HFB power is observed in the contra-lateral condition but not for the ipsi-lateral (error bars indicate ± 1 standard error, 15 trials per condition). Stimulus onset is at 0 s. Stimulus duration was 0.81 s and 0.5 s for the fMRI (**B**) and ECoG (**C**) measurements, respectively. Panels **D**–**F**, same as **A**–**C** for P2

HRFs corresponding to the contra-lateral and ipsi-lateral stimulation conditions were extracted from the voxels associated with each selected electrode (see “EcoG—analysis” section for electrodes selection and Fig. 3 for an example) via deconvolution using the hemodynamic response estimation toolbox (https://stat.columbia.edu/~martin/HRF_Est_Toolbox.zip) and using the finite impulse response model (Miezin et al. 2000). A total of 18 timepoints was estimated after each stimulus, corresponding to 15 s (equivalent to the inter-stimulus interval).

### ECoG–fMRI correspondence, analysis

The association between BOLD (PBR/NBR) and ECoG (HFB, alpha and beta power) at the single participant level was tested using a multivariate general linear model against the null hypothesis that no association was present. For each electrode that was selected (*n* = 33 and *n* = 27 in P1 and P2, respectively), the contralateral and ipsilateral response was derived from the deconvolved HRF and entered in a multivariate general linear model associated with ECoG mean power responses (HFB, alpha and beta). The same association across all the selected electrodes in P1 and P2 was tested using a generalized linear model with participants as a random effect (participant was coded as a categorical variable), accounting to some degree for variation between the two participants that took part in the experiment. Statistical analysis was performed in R (R Development Core Team 2010).

### ECoG—cortical localization along the visual hierarchy

We have obtained the location of the main ROIs along the visual hierarchy for each participant (P1 and P2) using the atlas provided in Wang et al. (2015) and the standardized surfaces in SUMA (Saad and Reynolds 2012).

For each participant, we have assigned each electrode to one surface-based ROI, based on the closest geodesic distance over the surface between the electrode and the surface-based ROI.

Wang et al. (2015) atlas provides within-ROI subdivisions between: ventral and dorsal V1/V2/V3, ventral/occipital areas 1 and 2 (Brewer et al. 2005), lateral occipital area 1 and 2 (Larsson and Heeger 2006), areas V3a and V3b (Press et al. 2001) and temporal occipital area 1 and 2 (Amano et al. 2009).

To facilitate visualization, we collapsed between ventral and dorsal V1/V2/V3, ventral/occipital areas 1&2, lateral occipital area 1&2 and temporal occipital area 1&2.

## Results

### ECoG responses to contralateral and ipsilateral stimulation

ECoG and fMRI data were acquired in separate sessions from two participants, while they were presented with a unilateral visual stimulation (Fig. 1). The participants underwent ECoG grid implantation for the purpose of epilepsy monitoring, and grids extended to healthy tissue in the occipital pole, the grids were placed to cover the calcarine fissure as well as the close surrounding cortex on the dorsal and lateral portion of the occipital pole (see Fig. 2A, B for a schematic of the electrode placement for each individual participant, P1 and P2).

**Fig. 4:**
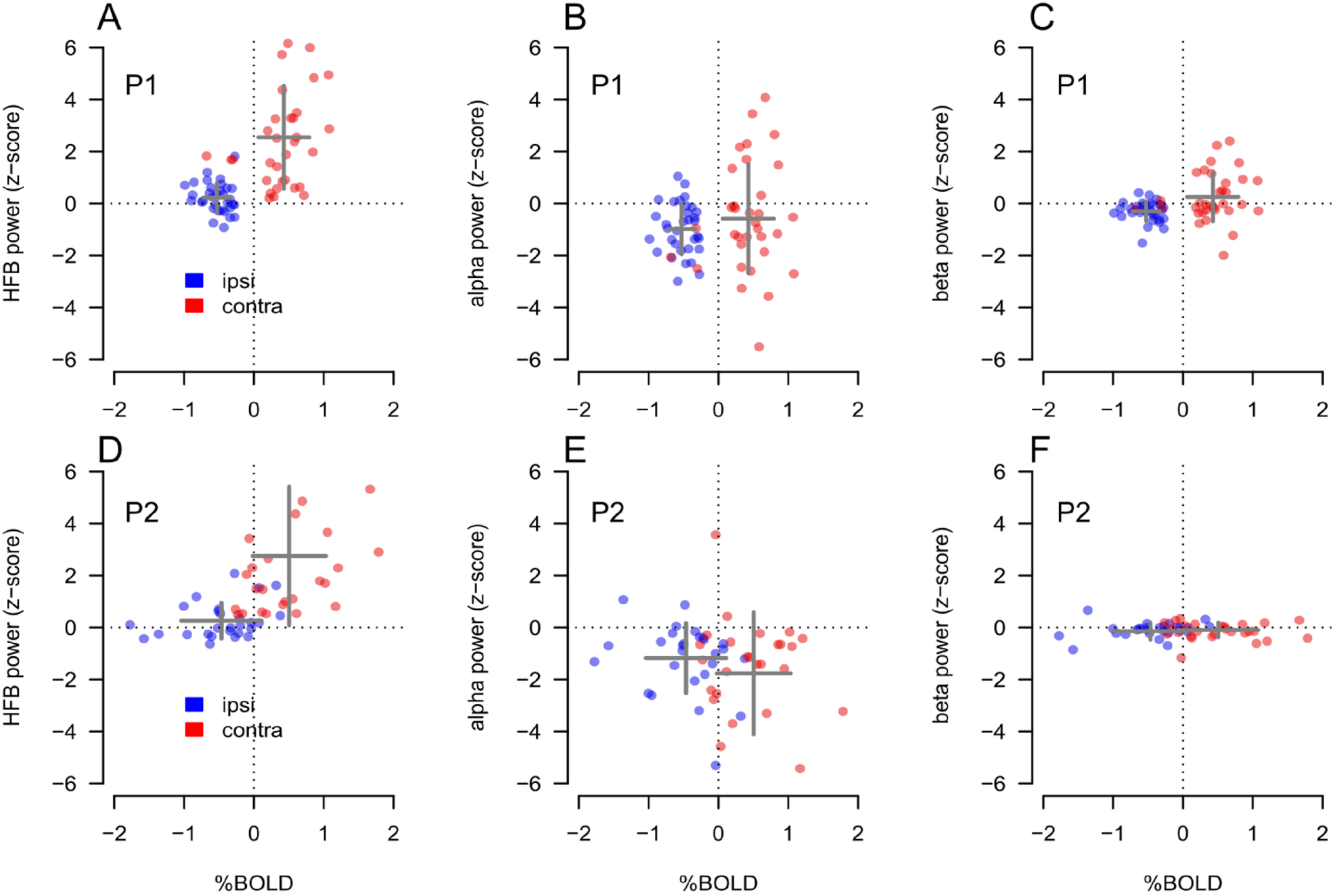
Summary results for both participants (P1&P2) and all selected electrodes; PBR and NBR % signal change was extracted from the local HRFs computed underneath each selected electrode. Panel **A**, data from P1, showing an increase in HFB power during the contra-lateral (red dots), but not the ipsi-lateral (blue dots), condition. The increase during the contra-lateral response is as associated with a positive BOLD response, each dot represents one electrode. On the other hand, the NBR associated with the ipsilateral condition is observed in the absence of HFB power responses, compared to baseline. Panel **B**, data from P1. Alpha power for the contra-lateral and ipsi-lateral conditions as a function of BOLD response for each electrode. NBR is associated with a decrease in alpha power. Panel **C**, data from P1. Beta power for the contra-lateral and ipsi-lateral conditions do not show a relationship as a function of BOLD response. It is important to note that, in Panel **C**, the distribution of blue points is shifted below zero along the vertical axes; average *z*-score across the selected electrodes = − 0.3. Panels **D**–**F**, data from P2, same as panels **A**–**C**; gray crosses in the plots indicate the average (center of the cross) and the standard deviation (width of the cross) of each %BOLD-power distribution

Contralateral visual stimulation during ECoG measurements did elicit a significant increase in the HFB power range (65–95 Hz) in *N* = 33 and *N* = 27 electrodes in P1 and P2, respectively. No significant responses were detected for the ipsilateral stimulation in this frequency range (one sample *t* test, *t*(32) = 2.24, *n.s.* and *t*(26) = 1.96, n.s, for P1 and P2, respectively, Bonferroni corrected, see Figs. 3C, F and 4A, D).

In both participants we observed a power increase in the HFB for the contralateral stimulation and a stimulus contrast reversal peak synchronous with the visual stimuli temporal frequency rate of 10 Hz and its harmonics (Fig. 2G, H), as previously reported (Gaglianese et al. 2017a, b). No significant power changes in the HFB range were detected for the ipsilateral stimulation for both participants (average power spectra across selected electrodes shown in Fig. 2G, H). All the *t* tests that follow regarding alpha and beta values responses were computed on the distribution of *z*-scores across all the selected electrodes that exhibit positive HFB power for contralateral visual stimulation (see “ECoG—analysis”).

For the contralateral visual stimulation, we measured an alpha power decrease for P2 among the selected electrodes (*t*(26) = − 4.53, *p* < 0.001, Bonferroni corrected). The same comparison did not reach statistical significance for P1 (average *z*-score across the selected electrodes = − 0.57, one sample *t* test, *t*(32) = − 1.57, *n.s,* Bonferroni corrected).

For ipsilateral visual stimulation we measured an alpha power decrease with respect to baseline in both participants (one sample *t* test, *t*(32) = − 5.71, *p* < 0.001 and *t* test, *t*(26) = − 4.53, *p* < 0.001, for P1 and P2, respectively, Bonferroni corrected, Fig. 4B, E).

Results for the beta band appeared to vary between participants. For P1, we measured a small but significant beta power decrease for the ipsi-lateral condition among the selected electrodes (average *z*-score across the selected electrodes = − 0.3, one sample *t* test, *t*(32) = − 4.51, *p* < 0.01 Bonferroni corrected, Fig. 4C) but not for the contra-lateral condition (average *z*-score across the selected electrodes = 0.25, one sample *t* test, *t*(32) = − 1.54, *n.s,* Bonferroni corrected). For P2 no significant power changes were observed in the beta-band for either condition (Fig. 4F).

### fMRI responses to contralateral and ipsilateral stimulation

Robust positive and negative BOLD fMRI signals were elicited in early and extra-striate visual cortex for the contralateral and ipsilateral stimulation conditions, respectively (P1, see Fig. 2A, C, D; P2, see Fig. 2B, E, F). The average shape of the positive and the negative hemodynamic response functions (HRFs) for cortical locations (voxels) significantly responding to the fMRI stimulus are displayed in Fig. 2D, F (global HRF, locations selected based on the contrast of contra-lateral minus ipsi-lateral condition). Overall in fMRI, contra-lateral stimulation elicited PBR, whereas the ipsi-lateral condition elicited NBR, as previously reported (Tootell et al. 1998; Gouws et al. 2014; Fracasso et al. 2018).

We analyzed the amplitude of the HRF extracted in the cortical locations corresponding to each selected electrode of each participant (*N* = 33 and *N* = 27 electrodes in P1 and P2, respectively, see “ECoG—analysis”). HRF for both contralateral and ipsilateral condition for a representative electrode of each participant was shown on Fig. 3A, B–D, E. During contralateral visual stimulation we observed PBR (one sample *t* test, *t*(32) = 6.72, *p* < 0.001 and *t*(26) = 4.57, *p* < 0.001, for P1 and P2, respectively, Bonferroni corrected, Fig. 4A, D), and NBR during ipsilateral visual stimulation (*t* test, *t*(32) = − 15.6, *p *< 0.001 and *t*(26) = − 4.58, *p* < 0.001 for P1 and P2, respectively, Bonferroni corrected see Fig. 4A, D).

### fMRI–ECoG correspondence

To investigate the correspondence between fMRI BOLD responses and the neurophysiological responses as measured by ECoG we compared the *z*-score responses in the HFB, alpha and beta band and the correspondent PBR and NBR amplitude peak responses derived from the deconvolved HRF in the fMRI measurements for each selected electrode (see “ECoG–fMRI co-localization and local HRF response”).

Contralateral stimulation elicited PBR in the voxels underneath the electrodes (Fig. 3B, E), which were associated with an HFB power increase for the duration of stimulation (500 ms), starting few milliseconds after stimulus onset (Fig. 3C, F). In the ipsi-lateral condition, NBR measured underneath the electrodes (Fig. 3B, E) were linked to HFB power just around zero for the duration of stimulation (Fig. 3C, F). A summary of the relationship between ECoG measurements in the HFB, alpha and beta band and the BOLD responses for each selected electrode is reported in Fig. 4.

To quantify the relationship between the neuronal responses and the BOLD responses we performed three separate multivariate analyses across all selected electrodes, for both the contralateral and ipsilateral stimulation. In the first analysis we tested individually for each participant whether PBR could be explained by HFB, alpha and beta power in the contra-lateral condition. In the second analysis we tested NBR against HFB, alpha and beta power in the ipsi-lateral condition (see Table 1, P1 and P2). In the third analysis we pooled all the recording sites in a single data set and accounted for individual participant variability using a generalized linear model with participants as a random factor (multilevel approach, see Table 1, P1 and P2).

**Table 1:** Association between BOLD and ECoG signal

	Beta coefficient	*t*-stat	*p* value
*Positive BOLD (contra-)*			
HFB (P1)	0.16	2.46	0.019*
Alpha (P1)	− 0.01	− 0.21	0.830
Beta (P1)	− 0.27	− 1.14	0.262
HFB (P2)	0.07	1.95	0.063
Alpha (P2)	− 0.10	− 2.40	0.024*
Beta (P2)	− 0.27	− 0.86	0.390
HFB (P1&P2)	0.08	2.87	0.005*
Alpha (P1&P2)	− 0.07	− 2.50	0.010*
Beta (P1&P2)	0.04	0.43	0.664
*Negative BOLD (ipsi-)*			
HFB (P1)	− 0.06	− 0.96	0.345
Alpha (P1)	− 0.01	− 0.24	0.807
Beta (P1)	− 0.01	− 0.12	0.904
HFB (P2)	0.16	1.10	0.282
Alpha (P2)	− 0.07	− 0.92	0.363
Beta (P2)	0.36	1.04	0.305
HFB (P1&P2)	0.04	0.54	0.589
Alpha (P1&P2)	− 0.07	− 1.68	0.096
Beta (P1&P2)	0.17	1.13	0.262

Multivariate general linear model results for contra-lateral and ipsi-lateral visual stimulation: beta coefficients, *t*-statistics and the associated *p* values are reported for both participants, individually (P1, P2; * indicate *﻿p* values are smaller than 0.05) as well as combined, using multilevel regression (P1&P2). Among the selected electrodes responding to visual stimulation (see “Materials and methods”), PBR is associated with increasing HFB and is negatively correlated with alpha power measured in the contralateral visual stimulation, while no significant association is observed for beta power. NBR is associated with decreases in alpha power but its amplitude does not appear to be linearly related with HFB, alpha or beta power in the ipsilateral condition (see also Fig. 4) among the selected electrodes

Given the small sample in the data set (two participants), we opted for reporting results at the individual participant level as well as with the multilevel approach.

We observed a positive linear relation between PBR and HFB responses for contralateral stimulation as well as a negative linear relationship with alpha power for the same condition among the selected electrodes (see Table 1). NBR in the ipsilateral condition did not show a clear linear association with ECoG measurements among the selected electrodes (Table 1).

### Electrode location and alpha activity in relation to BOLD

**Fig. 5:**
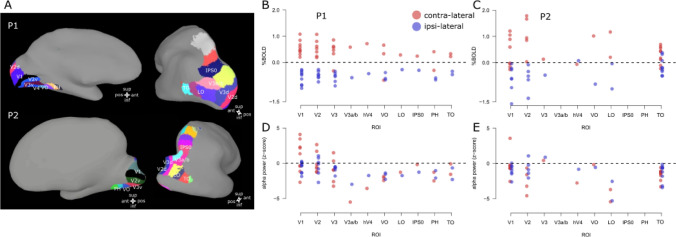
Electrode location along ROIs and activity (%BOLD and alpha power). Panel **A**: visually responsive ROIs based on the atlas provided by Wang et al. (2015) and the standardized surfaces in SUMA (Saad and Reynolds 2012). Panels **B**, **C**: %BOLD signal change along each ROI and experimental condition (contra-lateral and ipsi-lateral) for P1 and P2, each dot represents a single electrode. PBR is associated with the contra-lateral condition and NBR with the ipsi-lateral condition throughout the visual hierarchy. Panels **D**, **E**: alpha power (*z*-scored) along each ROI and experimental condition (contra-lateral and ipsi-lateral) for P1 and P2. Contralateral alpha power varies between as well as within cortical area. Ipsi-lateral alpha power on the other hand remains largely negative along the visual hierarchy

To provide a descriptive analysis of electrode location and activity along the visual hierarchy we have obtained the location of the main visually responsive ROIs for each participant (P1 and P2, see Fig. 5) using the atlas provided in Wang et al. (2015), collapsing any within-ROI subdivision and the standardized surfaces in SUMA (Saad and Reynolds 2012).

PBR is associated with the contra-lateral condition and NBR is associated with the ipsi-lateral condition, throughout the visual hierarchy. We observe limited NBR responses in contralateral visual stimulation that tend to be spread along the cortical hierarchy without a specific pattern. We interpret these responses as reflecting a mix of activity between surround suppression effects due to visual stimulation at the edge of population receptive field centers (pRF, Dumoulin and Wandell 2008; Fracasso et al. 2016a, b; Zuiderbaan et al. 2012), and ‘blood stealing’ from neighboring portions of stimulated cortex (Shmuel et al. 2006).

Alpha increases, when present, were observed in electrodes located in early visual cortex (V1–V3). Alpha decreases were detected across all ROIs for both stimulus conditions, although significant alpha decreases between the two participants (P1 and P2) were observed only in the ipsi-lateral condition.

### Control analysis

**Fig. 6:**
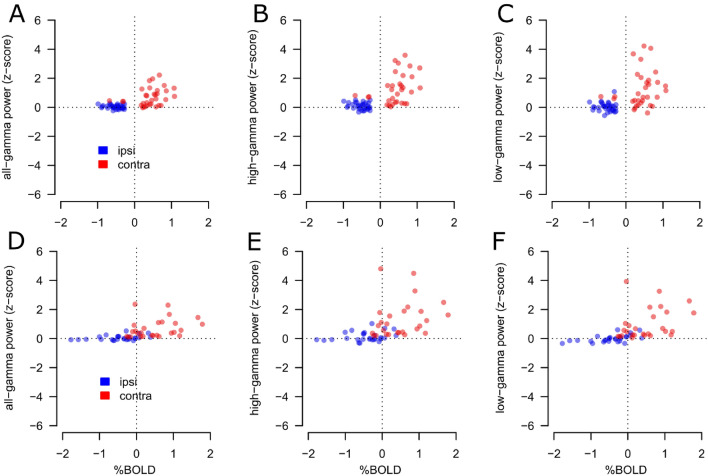
Summary results for both participants (P1&P2) and all selected electrodes for the low-gamma (31:64 Hz) high-gamma (65:130 Hz), and all-gamma (31:130 Hz); PBR and NBR % signal change was extracted from the local HRFs computed underneath each selected electrode. Panel **A**–**C**, data from P1. Gamma power results shows the contra-lateral (red dots) and ipsi-lateral (blue dots) conditions compared to the BOLD response for each electrode (each dot represents one electrode). Panel **A** shows the association between PBR and all-gamma power. On the other hand, NBR is observed in the absence of HFB power responses, compared to baseline. Panel **B**, same as **A**, for high-gamma power; Panel **C**, same as **A**, **B**, for low-gamma power; Panels **D**–**F**, data from P2, same as panels **A**–**C**, respectively; the association between PBR and gamma power and the NBR observed in the absence of HFB power responses is evident for each gamma power frequency range (low-gamma, high-gamma and all-gamma) and for each participant

To assess the robustness of the HFB results presented, we varied the frequency range of the measured HFB power changes in the contralateral and ipsilateral visual stimulation during the ECoG measurements. We opted for three different frequency ranges: low-gamma (30:64 Hz) high-gamma (65:130 Hz), and the all-gamma band (all-gamma: 30:130 Hz), see Fig. 6.

For each frequency range and participant, results were very similar to those observed with the frequency range 65–95 Hz reported in the section “ECoG responses to contralateral and ipsilateral stimulation”. Contralateral visual stimulation elicited a significant increase in the HFB power, while no significant responses were detected for the ipsilateral stimulation in this frequency range.

For P1, contralateral visual stimulation elicited a significant response in 33, 32 and 33 individual electrodes, for the all-gamma, high-gamma and low-gamma frequency ranges, respectively. No significant HFB responses were detected for the ipsilateral stimulation (one sample *t* test, *t*(32) = 1.61, n.s., *t*(31) = 2.18 n.s., and *t*(32) = 0.45 n.s., for the all-gamma, high-gamma and low-gamma, respectively.

For P2 results were virtually indistinguishable from P1, contralateral visual stimulation elicited a significant response in 27, 27 and 27 individual electrodes, for the all-gamma, high-gamma and low-gamma frequency ranges, respectively. No significant HFB responses were detected for the ipsilateral stimulation (one sample *t* test, *t*(26) = 1.61, n.s., *t*(26) = 2.05, n.s., and *t*(26) = − 0.12 n.s., for the all-gamma, high-gamma and low-gamma, respectively.

## Discussion

We measured fMRI and ECoG responses elicited by visual stimulation to characterize the correspondence between negative BOLD responses and neurophysiological activity in human participants. We applied a task able to elicit both PBR and NBR in response to contralateral and ipsilateral visual stimulation (Tootell et al. 1998; Gouws et al. 2014; Fracasso et al. 2018). The paradigm allowed us to record responses dominated by PBR and NBR from the *same* portion of visual cortex (Figs. 1, 2).

For the selected electrodes responding to visual stimulation (see “ECoG—analysis”), our main results are the positive correlation between PBR and HFB power increase, and, in the ipsi-lateral condition, the presence of NBR in the absence of HFB power changes measured by ECoG. We obtained similar results for the contralateral and ipsilateral visual stimulation when varying the frequency range of the measured HFB power (low-gamma, high-gamma and a combination of the two).

Interestingly, an increase in alpha power was detected only in electrodes located in early visual regions V1, V2 and V3 independently on the stimuli condition. This in in line with the surround-suppression effect seen in striate cortex (Harvey et al. 2013).

Surprisingly, in the ipsilateral condition NBR was observed in presence of negative alpha power, consistent with the idea that alpha activity reflects functional inhibition (Jensen and Mazaheri 2010; Palva and Palva 2011).

### Negative BOLD

Several processes have been proposed to account for NBR and it is likely that different mechanisms are responsible, depending on the specific experimental conditions (Goense et al. 2012a, b). Initially, NBR was believed to be the consequence of a decrease in cerebral blood volume (CBV) due to neighboring positive BOLD activity, referred to as the blood-stealing effect (Harel et al. 2002). This mechanism can account for negative BOLD signal located close to positive BOLD signal.

Further experiments have shown that NBR could be observed following a reduction of neuronal activity and a decrease in cerebral blood flow (CBF) (Shmuel et al. 2002, 2006; Devor et al. 2008; Boorman et al. 2010; Goense et al. 2012a, b), suggestive of a completely different neurovascular coupling mechanism compared to the blood-stealing effect. In our experiment, PBR and NBR were extracted from the same location in the same hemisphere, which agrees with the latter.

In the literature, NBR are also observed in the sensory-motor domain. During unilateral hand movement, the contralateral cortex is activated, showing increases in HFB power as well as PBR. However, in the ipsilateral side NBR is routinely observed (Devor et al. 2008; Kastrup et al. 2008; Schafer et al. 2012). Interestingly, in this case, ipsilateral NBR co-occurs with neuronal excitation as well as trans-callosal inhibition (Nass 1985; Allison et al. 2000). This pattern of results has been attributed to active inhibition by inhibitory GABA interneurons in the ipsilateral cortex (Devor et al. 2008; Kastrup et al. 2008; Schafer et al. 2012). The combination of excitation and inhibition in the ipsilateral side could vary and was dependent on the task demands (i.e., the movement rate). However, recent evidence suggests that activation of inhibitory neurons can increase local cerebral blood flow independently of net ongoing neuronal activity (Anenberg et al. 2015).

Moving to the visual domain, ipsilateral NBR has been observed previously (Tootell et al. 1998; Gouws et al. 2014; Fracasso et al. 2018), and was also shown to be dependent on task demand, with attention-demanding tasks on the contralateral stimuli leading to stronger ipsilateral NBR (Gouws et al. 2014). Overall, a variety of different sources have been identified to be responsible for the observation of NBR and these sources have been shown to differ depending on the exact task demands.

### BOLD—ECoG correspondence during contralateral stimulation

As expected, our results showed a positive correlation between PBR and HFB responses elicited by contralateral visual stimulation among the selected electrodes. PBR is known to be coupled with high-frequency electrophysiological responses observed in intra-cortical recordings (Logothetis et al. 2001), ECoG (Lachaux et al. 2007; Siero et al. 2014; Gaglianese et al. 2017a, b), MEG (Brookes et al. 2005) and EEG (Ball et al. 2008; Mulert et al. 2010). Moreover, PBR were correlated with decreased alpha power in the contralateral condition, consistent with multiple studies combining fMRI and EEG (Goldman et al. 2002; Laufs et al. 2003; Feige et al. 2005; Scheeringa et al. 2009) and with the idea that alpha activity reflects functional inhibition (Jensen and Mazaheri 2010).

### BOLD—ECoG correspondence during ipsilateral stimulation

In the current investigation NBR measured during ipsilateral visual stimulation were accompanied with an absence of HFB power response measured by ECoG. In contrast, an earlier study showed an association between a decrease in BOLD signal and decreased neural firing rate in multi-unit activity below spontaneous activity (Shmuel et al. 2006; Boorman et al. 2010). The difference between our and earlier findings could be explained by the type of electrodes adopted in ECoG compared to animal neurophysiology. Indwelling electrodes are used in the latter case, which measure responses from one or several neurons, whereas in the former electrodes are placed over the brain surface, which sample from several hundreds of thousands of neurons. If a relatively small number of neurons exhibits a decrease in firing rate, this may not be observed when measuring with surface electrodes.

A significant decrease in alpha power in response to ipsilateral stimulation was measured across responding electrodes, in both participants. Although no linear association was found between NBR and alpha power responses, this alpha power decrease was concomitant with NBR (Fig. 4B, E).

We can speculate about the neuronal underpinnings of the alpha power decrease. The electrodes in the ipsilateral hemisphere are pooling from neuronal populations with a corresponding population receptive field. It could be argued that the suppressive surround of this population receptive field might extend over the participant’s midline, into the contralateral visual field. Thus, our contralateral stimuli might stimulate the suppressive portion of the corresponding population receptive field, yielding NBR (Zuiderbaan et al. 2012). However, in the scenario just described we would expect an increase in alpha power as reported in Harvey et al. (2013). This is not compatible with our data, as we observed a decrease in alpha power instead.

Alternatively, literature from the sensory-motor domain shows that unilateral hand movement induces NBR in the ipsilateral motor cortex, which could arise from the activity of inhibitory GABA interneurons that are actively suppressing neuronal activity via trans-callosal connections (Schafer et al. 2012).

Moreover, Mullinger and colleagues used median nerve stimulation and EEG recordings showing ipsilateral decreases in BOLD signal concomitant with decreases in the alpha power range (8–13 Hz; Mullinger et al. 2014). This may suggest that activity in inhibitory GABA interneurons is related to the ipsilateral alpha power decreases observed by Mullinger and co-workers.

Based on the results obtained in the sensory-motor domain (Schafer et al. 2012; Mullinger et al. 2014) we speculate that GABAergic inhibition might drive the alpha power decrease we observed in the ipsilateral condition. Note that this decrease was observed for the same electrodes that showed a response in HFB and an associated decrease in NBR. However, the data available does not allow us to draw conclusions about the underlying GABA mechanism at play.

ECoG–fMRI research can positively affect also non-invasive EEG findings. EEG can provide large-scale information about electrophysiological and hemodynamic measurements, while ECoG can provide evidence on a meso- and micro-scale, showcasing tuned and localized neuronal responses and their link to behavior (Schölvinck et al. 2010; Marino et al. 2019; Seeber et al. 2019).

## Limitations

Only electrodes showing significant positive HFB responses for contralateral stimulation were selected for further analysis (*n* = 33 and *n* = 27 in P1 and P2, respectively, see section “ECoG—analysis”). These selection criteria were necessary in the current study as we set to investigate BOLD responses on those locations, where ECoG showed a reliable HFB response. The introduction of these selection criteria is a limitation that we acknowledge, and it is largely due to the limited available runtime to acquire fMRI and ECoG data with our participants.

A further limitation of this study is the low number of participants (*n* = 2). We mitigate this by showing individual data and individual-based analysis, using the number of electrodes per participant as statistical unit. We aim to get a significant result in every subject (Vansteensel et al. 2016). This is in contrast with studies, where effects only reach significance when averaging across subjects. Therefore, the number of subjects is needed to build confidence that your effect is reproducible across subjects, i.e., subjects are replication units not measurement units. For discussion in defense of small sample sizes (provided strong measurements) see (Normand 2016; Smith and Little 2018).

Reporting individual data and individual-based analysis indicates that our results are not dependent on the number of subjects included in the analysis. Based on these results, we consider the possibility of a false negative response in the ipsilateral condition unlikely, because contralateral and ipsilateral stimuli were presented within the same task, and we see a contralateral response in the same electrodes, where the ipsilateral responses were absent.

Moreover, ECoG measurements are a unique tool to directly measure task-related neural activity in the human brain and provide valuable information on brain processing and function. The high sensitivity of ECoG allows to infer brain processing in small cohort of patients and ECoG single subject cases have been determined to be informative before (Harvey et al. 2013; Van der Stigchel et al. 2019; de Jong et al. 2020; Gaglianese et al. 2020).

## Conclusions

In this study we expanded the investigation of NBR using fMRI and ECoG in human participants. Our findings show that NBR are associated with an absence of power increases of HFB activity and spatially co-localized alpha power decreases, strengthening the view that the NBR is connected to underlying decreases in neural activity. We speculate on the potential contribution of GABAergic inhibition to the observed negative BOLD response.

## Data Availability

All data analysed in this paper can be obtained by emailing a request to the first author.

## Code availability

The analysis code can be obtained by emailing a request to the first author.
